# The complete mitochondrial genome of *Leptopilina syphax* (Hymenoptera: Figitidae)

**DOI:** 10.1080/23802359.2020.1845106

**Published:** 2021-01-06

**Authors:** Qichao Zhang, Xian Zhang, Ying Wang, Jiani Chen, Sicong Zhou, Lan Pang, Zhizhi Wang, Yiping Wang, Xuexin Chen, Jianhua Huang

**Affiliations:** a Institute of Insect Sciences, College of Agriculture and Biotechnology, Zhejiang University, Hangzhou, China; b Ministry of Agriculture Key Lab of Molecular Biology of Crop Pathogens and Insect Pests, Zhejiang University, Hangzhou, China; c Key Laboratory of Biology of Crop Pathogens and Insects of Zhejiang Province, Zhejiang University, Hangzhou, China; d College of Forest and Biotechnology, Zhejiang Agricultural and Forestry University, Lin’an, China; e State Key Lab of Rice Biology, Zhejiang University, Hangzhou, China

**Keywords:** *Leptopilina syphax*, parasitoid, mitochondrial genome

## Abstract

*Leptopilina syphax* (Hymenoptera: Figitidae) is a newly recorded species of parasitic wasp, and it attacks the larval stage of Drosophilidae, mainly the *Drosophila* species. Few works have been done in the basic study of *L. syphax*, including the data of mitochondrial genome. In this study, the complete mitochondrial genome of *L. syphax* (GeneBank accession number: MT649407) was sequenced using Illumina HiSeq X Ten system. The mitochondrial genome is 15,882bp long and comprises 13 protein-coding genes, 2 ribosomal RNA genes and 22 transfer RNA genes. Meanwhile, 26 genes are in majority strand, and the remaining 11 genes are in minority strand. The overall base composition is 41.7% for A, 6.0% for G, 13.6% for C, and 38.7% for T, with an A + T content of 80.4%, respectively. We also performed a phylogenetic analysis with other known mitochondrial genomes of some parasitic wasps. The results show that *L. syphax* is closely related to *L. boulardi*, which is another *Drosophila* parasitoid.

Complete mitochondrial genomes are useful tools for molecular evolution analysis and they have been wildly used to study phylogenetic relationship in both vertebrates and invertebrates (Ingman et al. 2000; Broughton et al. 2001; Mao et al. 2015). Mitochondrial genomes are treated as molecular markers for phylogenetic studies with many beneficial characteristics, such as lack of recombination, comparatively high mutation rate and the strictly maternal inheritance (Boore 1999). *Leptopilina syphax* is a newly recorded species of parasitic wasp and it attacks the larval stage of Drosophilidae, mainly the *Drosophila* species. Meanwhile, little has been done for its phylogenetic and evolution analysis.

*Leptopilina syphax* was collected by a net-trap method on May 2018 at Taizhou (28°50′N, 120°34′E), Zhejiang, China. The specimen (ZJUHJH_003) was stored in 100% ethanol and kept in the Parasitic Hymenoptera Collection of Institute of Insect Sciences, Zhejiang University. The DNA of *L. syphax* was isolated and purified using the standard phenol-chloroform method. The mitochondrial genome of *L. syphax* was sequenced by Illumina HiSeq X Ten system with the 150 paired-ends reading strategy. It was further annotated using the Geneious (version 11.0.4) and MITOS Web Server (Bernt et al. 2013).

The length of the complete mitochondrial genome of *L syphax* is 15,882 bp, and it contains 37 genes which includes 13 protein-coding genes (PCGs), 22 transfer RNA genes (tRNAs), 2 ribosomal RNAgenes (rRNAs), and a putative control region (CR). Further analysis revealed that 26 genes are encoded on the majority strand, while the remaining 11 genes are encoded on the minority strand. The gene order in the *L. syphax* mitochondrial genome is very similar with another *Leptopilina* species, *L. boulardi* (Oliveira et al. 2016). The overall base composition is 41.7% for A, 6.0% for G, 13.6% for C, and 38.7% for T, with an A + T content of 80.4%. Three start codons for PCGs are used: ATA (*nad2*, *nad1* and *nad5*); ATT (*cox1*, *atp8*, *cox3*, *nad4l* and *cob*); ATG (*cox2*, *atp6*, *nad3*, *nad4* and *nad6*). 10 PCGs use a TAA stop codon and 3 PCGs (*cox3*, *nad3* and *cob*) use a TAG stop codon. The 22 tRNAs genes vary from 58 bp to 73 bp in length, and the secondary structure of tRNAs are typical clover-leaf structures as with other insects. The *rrnL* was located next to *rrnS*, and they both were located between *trnD* and *trnC*. The length of *rrnL* and *rrnS* was 1316 bp and 817 bp, respectively.

Till now, 50 hymenopterous parasitoid species have been founded to attack Drosophilid species worldwide, and the majority of which are in the following four families including Braconidae, Figitidae, Diapriidae and Pteromalidae. We performed the phylogenetic analysis of *L. syphax* with some other parasitoids from the four families and with *Megaphragma amalphitanum* (Trichogrammatidae) and *Ibalia leucospoides* (Ibaliidae) as well, which are closely related with Pteromalidae and Figitidae, respectively. (Oliveira et al. 2008, 2016; Li et al. 2016; Tang et al. 2019; Zhang, Li, et al. 2020; Zhang, Pan, et al. 2020). The sequences were aligned using MAFFT v7.271, and the phylogenetic tree was constructed by CIPRES (https://www.phylo.org/) using RAxML-HPC2 on XSEDE with bootstrap 1000 and MrBayes on XSEDE (Figure 1). Phylogenetic analysis showed that *L. syphax* is closely related to *L. boulardi*, another *Drosophila* parasitoid in *Leptopilina* genus. This study would further clarify our understanding of the phylogenetic relationship of the Figitidae family.

**Figure 1.**
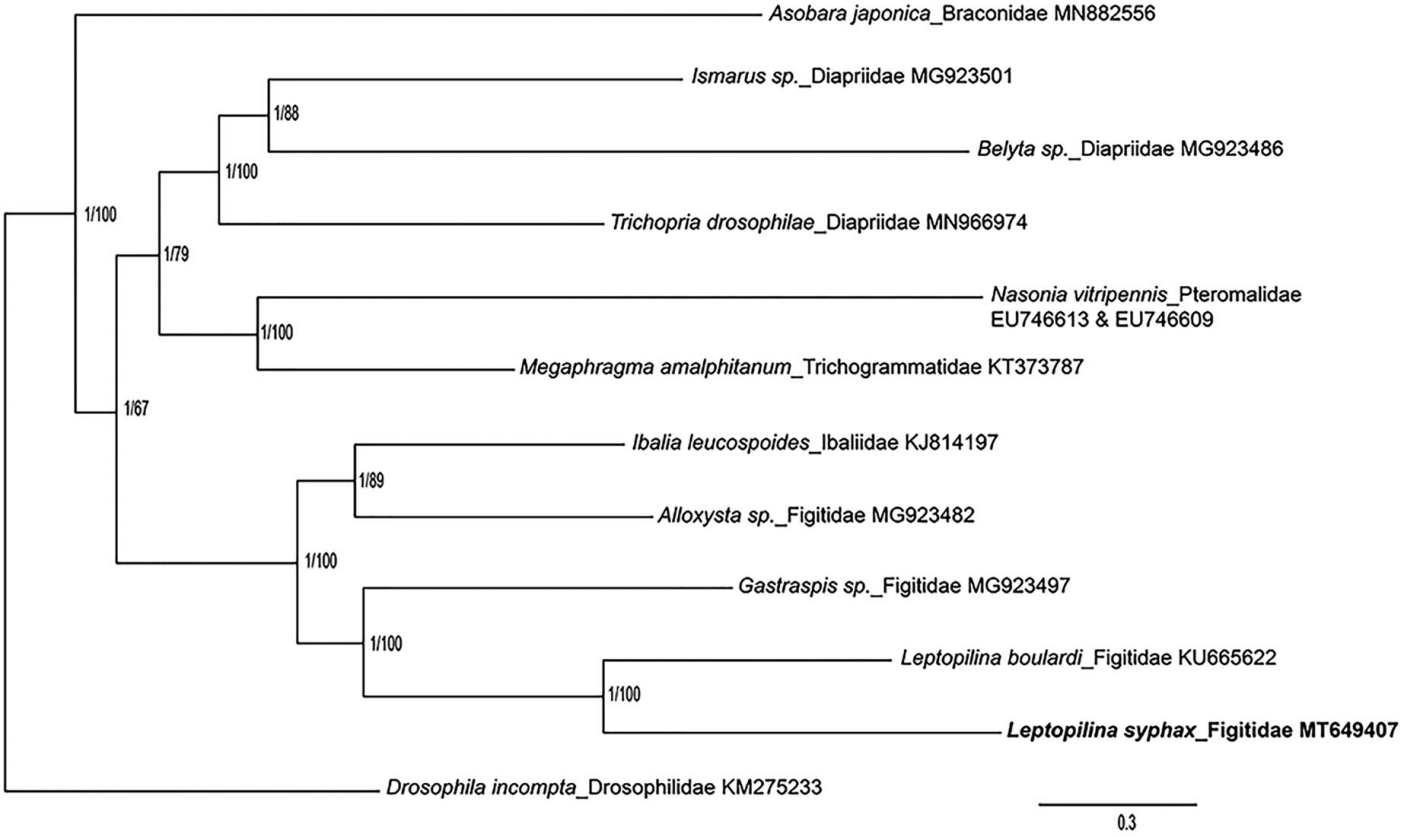
Phylogenetic relationships among selected parasitoids inferred from nucleotides of 13 PCGs and 2 rRNAs using Bayesian and maximum-likelihood (ML) methods (GenBank accession numbers provided). The Bayesian posterior probabilities (PP) and bootstrap support (BS) are marked beside the nodes. *Drosophila incompta* was set as outgroup.

## Data Availability

The data that support the findings of this study are openly available in GenBank of NCBI at https://www.ncbi.nlm.nih.gov, reference number MT649407.
